# Supplementary material for: Seeing the Forrest through the trees: Oculomotor metrics are linked to heart rate
Source: PLoS One. 2022 Aug 2;17(8):e0272349. doi: 10.1371/journal.pone.0272349 (PMC9345484; doi:10.1371/journal.pone.0272349)
Supplement: S1 Table — (DOCX) [file pone.0272349.s001.docx]

## S1 Table. List of statistical descriptors.

1. Mean
2. Variance
3. Skew
4. Kurtosis
5. Range
6. 10^th^ Percentile
7. 90^th^ Percentile
8. Interquartile Range
9. Absolute Mean Deviation
10. Energy
11. Root Mean Square
12. Entropy
13. Uniformity
